# Neuroectodermal Diseases: A Comparative Case Report Study

**DOI:** 10.7759/cureus.40349

**Published:** 2023-06-13

**Authors:** John Paul M Cruz, Michelle Sy

**Affiliations:** 1 Neurology, Quirino Memorial Medical Center, Quezon City, PHL

**Keywords:** mtor, ras, pigmentary mosaicism, hemimegalencephaly, neurocutaneous disease, seizure, developmental delay

## Abstract

Neuroectodermal disease involves abnormalities that arise from the ectodermal origin, such as the nervous system, eyeball, retina, and skin. Due to the rarity of the disease, it is often underdiagnosed or misdiagnosed. In this study, the researcher presents two cases of pediatric patients with no fetomaternal complications who presented with focal seizures as their initial complaint.

During the examination, varying skin color pigmentation and an abnormal neurophysical examination were observed. Cranial imaging showed hemimegalencephaly and voltage asymmetry on EEG. Skin biopsy was performed on both cases, which revealed basketweave orthokeratosis.

The combination of a triad of intractable epilepsy, developmental delay, and cutaneous lesion prompted the consideration of a neuroectodermal disease. The study shows two cases of hypomelanosis of Ito and nevus syndrome, both of which may be due to mTOR and RAS pathways, respectively.

## Introduction

Neuroectodermic disease involves abnormalities that arise from ectodermal areas, such as the nervous system, the eye, the retina, and the skin [1].

Hypomelanosis of Ito and linear nevus sebaceous syndrome are both neuroectodermic diseases, and they occur with an incidence of one in 8,000-10,000 people in the general population and every one in 1,000 live births [2]. The clinical findings may be subtle at first until seizure and developmental delay occur, which are present in 90% of cases with hypomelanosis of Ito and almost two-thirds of cases of linear nevus sebaceous syndrome [3]. At present, there are few cases in the literature that have been reported.

Indeed, this study will contribute by adding more information to the present knowledge of neuroectodermal diagnosis and help in early diagnosis by looking at associated findings.

Objectives

This study aims to present two pediatric cases presenting with seizures and diagnosed with neurocutaneous disease. Additionally, this work aims to highlight the similarities and differences between these cases, as well as their pathophysiology, diagnostics, and treatment.

## Case presentation

Case 1

An eight-year-old female, right-handed child, born to a G2P2 female (2002) presented to the emergency department due to several episodes of seizures. The patient has had focal epilepsy since she was eight months old, with stiffening of all the extremities, upward rolling of the eyes, and head deviation to the left. These seizures tend to occur three times per day and last up to one minute. The patient had no history of CNS infections or trauma. The patient was prescribed oxcarbazepine (18 mg/kg/day), which she tolerated well.

Two days prior to the presentation, the patient was not able to take her anti-seizure medication due to it being unavailable. On the same day, the patient had multiple breakthrough seizures presented as twitching on the left side of her face. She was rushed to the emergency department, given levetiracetam at 35 mg/kg/day, and then discharged. However, the seizure persisted, but this time it lasted for 30 minutes, came back again after a day, and was managed as status epilepticus.

**Figure 1:**
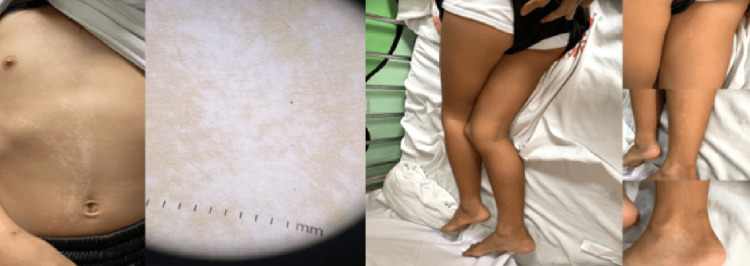
Hypopigmented cutaneous lesion on the right side of the trunk, thigh, and leg

**Figure 2:**
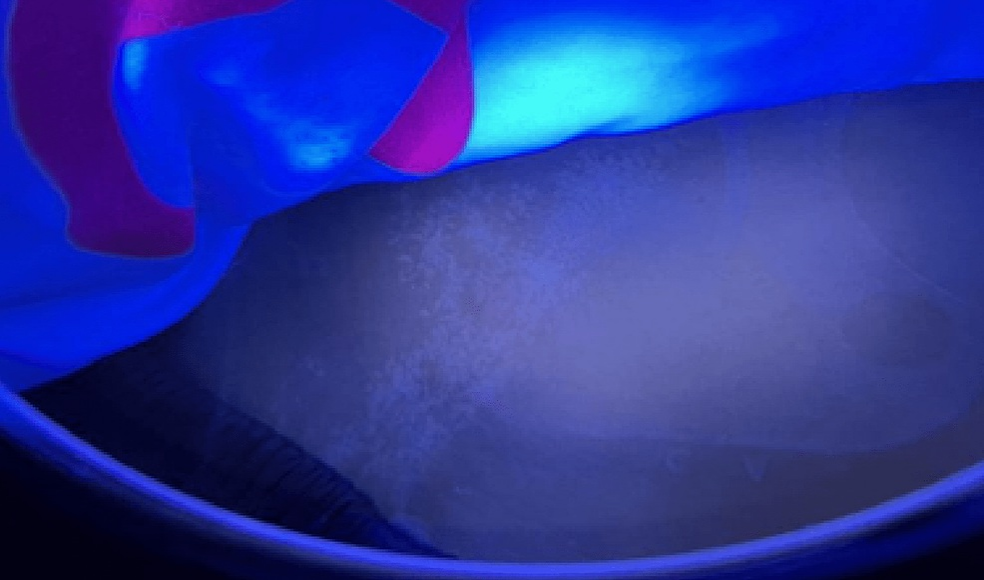
Enhancement of pattern on Wood’s lamp

On examination, it was noted that the patient had cutaneous lesions consisting of multiple, ill-defined, hypopigmented macules and patches in linear configurations on the right side of her chest, abdomen, posterior thigh, and leg (Figure 1), which showed enhancement of pattern under Wood's lamp (Figure 2).

**Figure 3:**
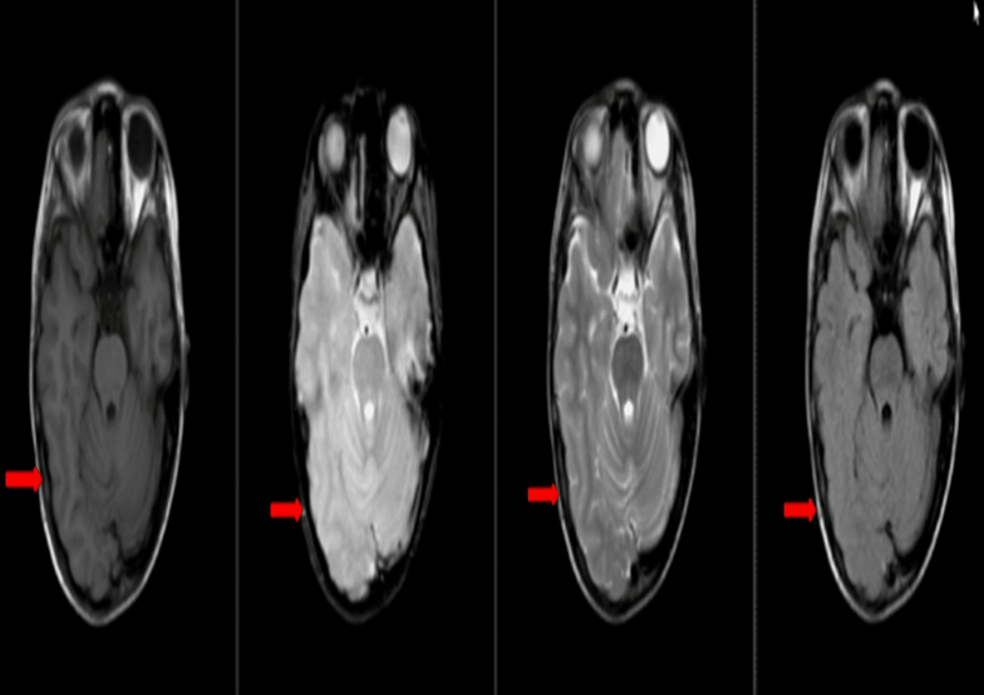
Red arrows on T1, T1 contrast, T2, and fluid-attenuated inversion recovery according to arrangement showing hemimegalencephaly on the right

A cranial MRI with IV contrast and an EEG were conducted. The MRI showed hemimegalencephaly on the right cerebral hemisphere, as indicated by a red arrow (Figure 3).

**Figure 4:**
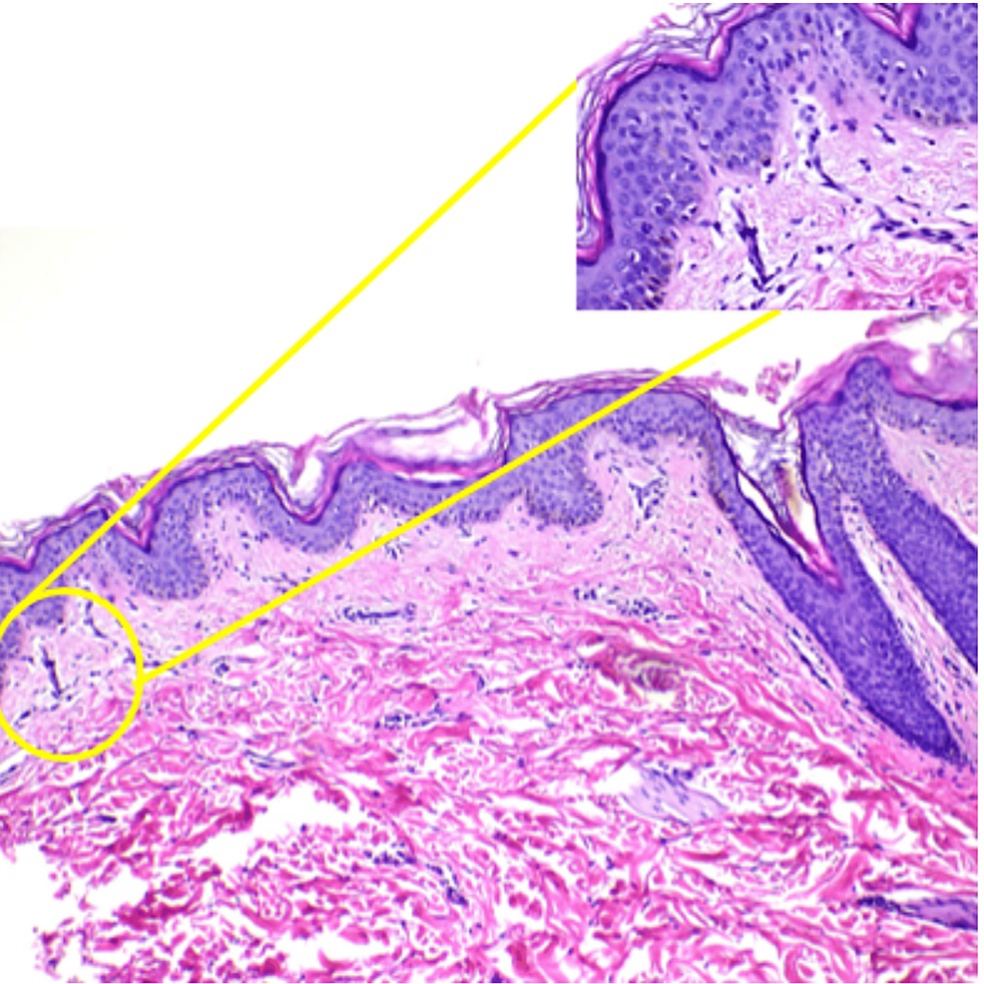
Skin biopsy on the hypomelanotic skin lesion with post-inflammatory pigment alteration

A skin biopsy was done on the hypomelanotic skin lesion, and the results were consistent with post-inflammatory pigment alteration. The magnified view as traced by the yellow line showed basketweave orthokeratosis, with decreased melanocytes and melanin at the basal layer. In the dermis, there were few perivascular lymphocytic infiltrates, which are suggestive of hypomelanosis of Ito (Figure 4).

In the EEG, there was an abnormal voltage asymmetry with voltage attenuation over the right hemisphere and frequent right frontocentrotemporal epileptiform discharges.

Serum electrolytes, a complete blood count, and an ECG for cardiac evaluation were unremarkable.

Case 2

A nine-month-old female presented to the emergency department due to six episodes of stiffening of the right upper and lower extremities.

The patient’s medical history started when she was one month old. She was apparently well until she was noted to experience six episodes of head deviation to the right and stiffening of the right upper and lower extremities lasting for 30 seconds to two minutes with two-hour intervals between the episodes. The episodes were present both in wakefulness and sleep. The patient was initially prescribed valproic acid but developed skin allergies; therefore, the patient was instead prescribed levetiracetam 60 mg/kg/day, and lacosamide 2 mg/kg/day was later added.

Subsequently, the patient experienced breakthrough seizures, triggered by infection at a frequency of up to three/day, lasting less than one minute. Her last admission was on July 20, 2022, due to a provoked seizure secondary to pneumonia.

In terms of the patient's full history, the patient was born to a 35-year-old G3P3 female (2013) at 35 weeks and four days of gestation and delivered via cesarean section secondary to axilloabdominal cystic hygroma. A prenatal check-up was performed at six months of gestation, and the mother had a regular intake of supplemental vitamins.

At birth, the patient was acrocyanotic with a good cry, some flexion, frontal bossing, a flat nasal bridge, and non-bulging fontanelles. An axilloabdominal cystic hygroma measuring 18 x 24 cm was also present and subsequently operated on.

At seven months, the patient was referred to a developmental pediatrician since developmental milestones were delayed compared to siblings or peers. At that time, the patient could roll over but had poor head control, was unable to pull up to sit, babbled, and was not searching for dropped objects.

**Figure 5:**
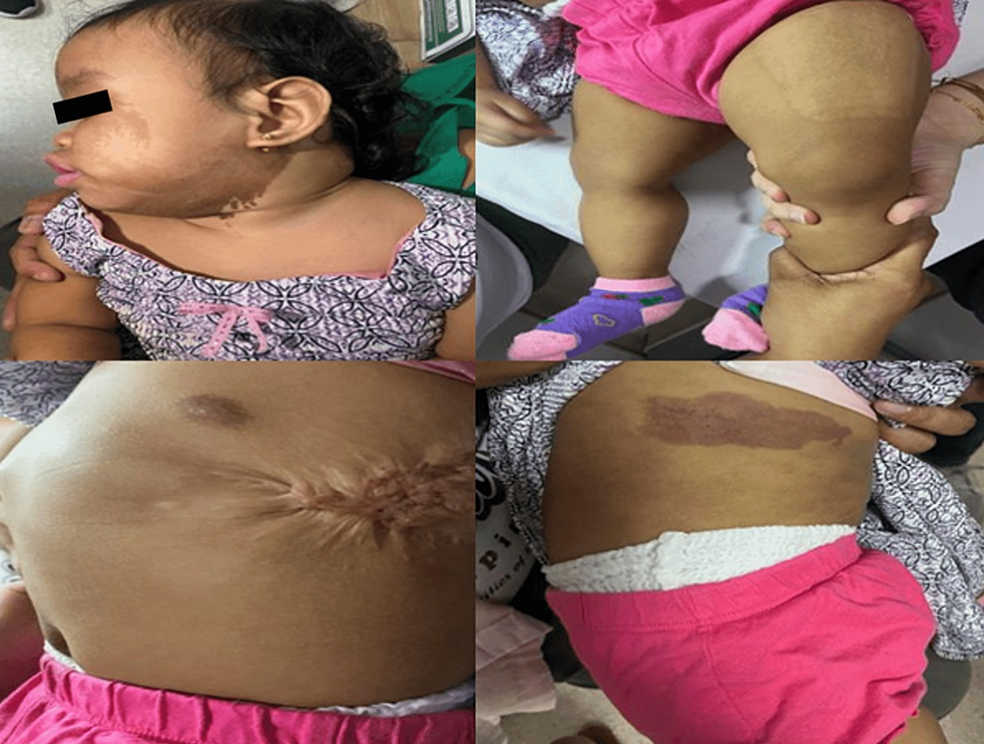
Multiple, well-defined, hyperpigmented light brown patches involving the central forehead, extending to the left cheek and neck with larger facial and body features on the left

In the most recent admission at nine months old, the patient’s physical examination revealed multiple, well-defined, light brown patches involving the central forehead that extended to the cheek and neck, as well as larger facial and body features on the left (Figure 5).

A neurophysical examination revealed an awake and alert patient who could turn her head in response to visual and auditory cues and showed spontaneous movement of all the extremities. The pertinent findings were the bilateral persistence of plantar, palmar, and Moro reflexes.

**Figure 6:**
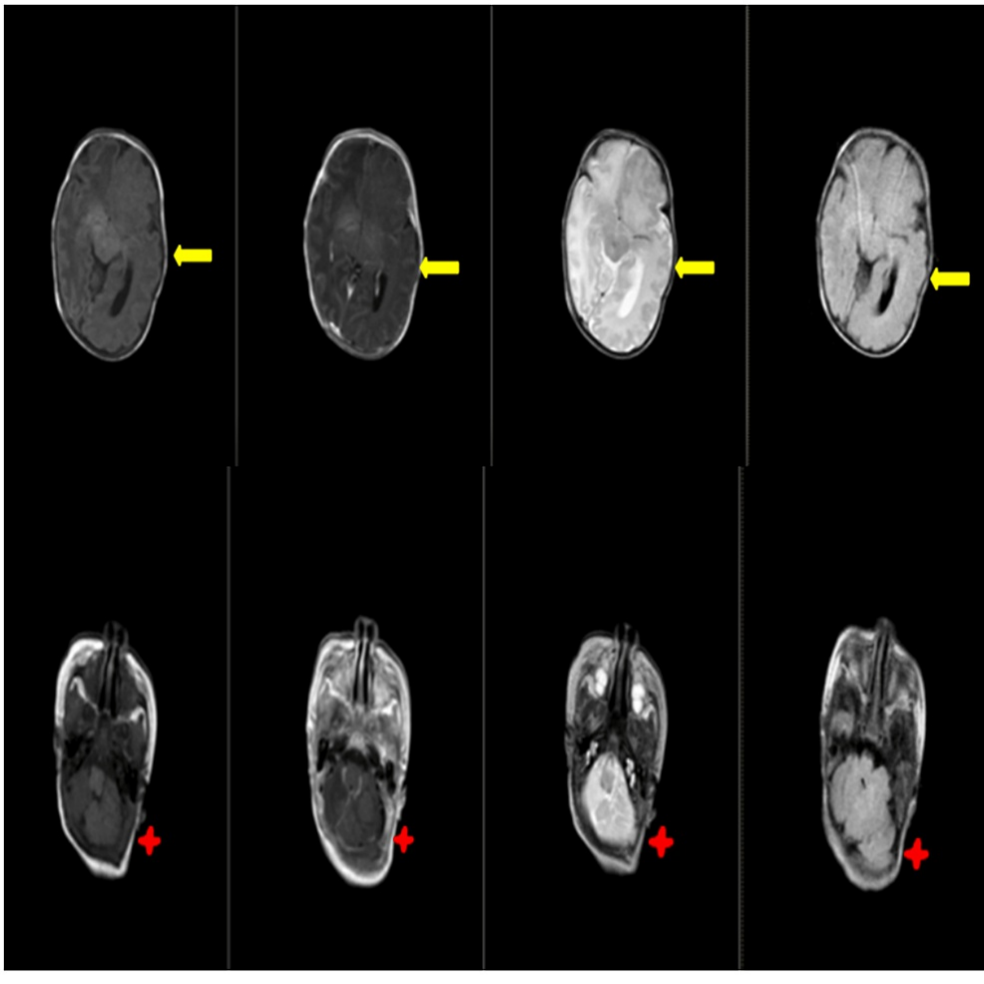
Yellow arrows in T1, T1 contrast, T2, and fluid-attenuated inversion recovery according to arrangement show left hemimegalencephaly associated with polymicrogyria-pachygyria, gray matter heterotropias, and lateral ventricular enlargement, while the red four-point star shows a concomitant ipsilateral cerebellar enlargement

The patient had a chromosomal analysis, which revealed normal results. Diagnostics such as cranial MRI with IV contrast, a skin biopsy, and an EEG were also performed. In the cranial MRI with IV contrast, there was the presence of left hemimegalencephaly associated with polymicrogyria-pachygyria, gray matter heterotropias, and lateral ventricular enlargement seen with yellow arrows and concomitant ipsilateral cerebellar enlargement seen with a red four-point star (Figure 6).

**Figure 7:**
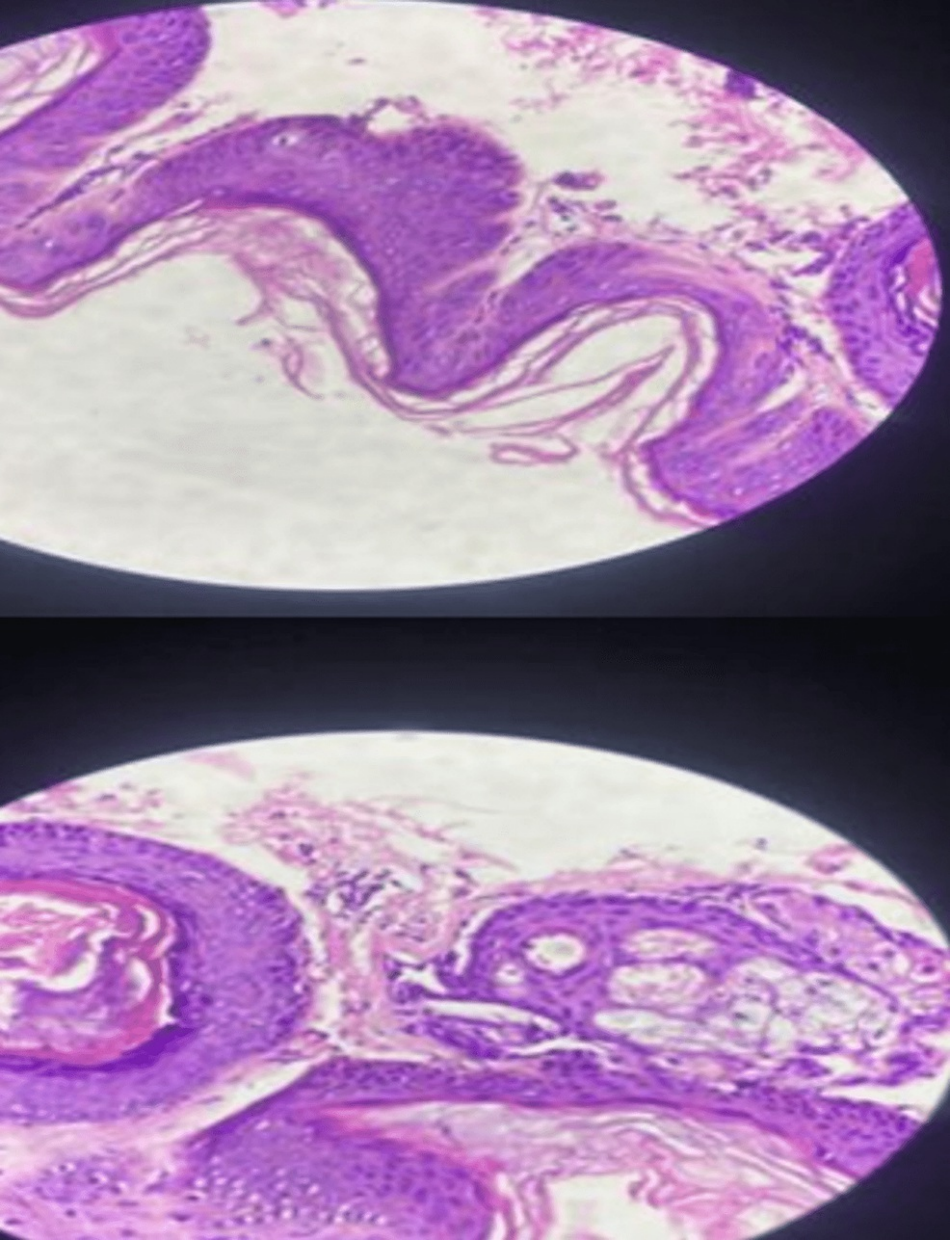
Basketweave orthokeratosis, papillomatosis, and hypergranulosis, with focal koilocytic change

A skin biopsy was done on the hyperpigmented skin lesion and the results showed basketweave orthokeratosis, papillomatosis, hypergranulosis with focal koilocytic change, irregular epidermal hyperplasia with a flat base, focal spongiosis, and a superficial perivascular lymphocytic infiltration, consistent with epidermal nevus (nevus verrucosus) (Figure 7).

In the EEG, there was high voltage background activity over the left side of the brain with occasional focal epileptiform discharges over the left frontotemporal and centro-parietal area, which were suggestive of focal epilepsy.

Serum electrolytes, a complete blood count, and an ECG for cardiac evaluation were unremarkable.

## Discussion

Hypomelanosis of Ito

Case 1 presents an example of hypomelanosis of Ito due to the presence of seizure, abnormal neurologic findings, and hypopigmented skin lesions. Hypomelanosis of Ito, previously named incontinentia pigmenti achromians, is a rare neurocutaneous disease with multisystem involvement. The cutaneous manifestation is characterized by unilateral or bilateral macular hypopigmented or hyperpigmented whorls, streaks, and patches that sometimes follow the line of Blaschko [3]. The cutaneous manifestation does not follow the nerves, vessels, or lymphatics; however, it may represent a clinical expression of a genetically programmed clone of altered cells, which are perhaps first expressed during embryogenesis [4]. However, the cause of the pattern of Blaschko lines is still unknown [4]. A close differential diagnosis is tuberous sclerosis complex, in which the hypopigmented patch (an ash leaf spot that is usually oval in shape and has regular borders) appears during the first year of life, mainly on the trunk or extremities [3].

Neurological manifestations are present in 90% of cases of hypomelanosis of Ito. The cohort study of Carmignac et al., which included patients with phenotypes of hypopigmented skin lesions in a mosaic pattern, showed that certain proportions of these patients experienced intellectual delay (80%), epilepsy (67%), macrocephaly (53%), hemimegalencephaly/megalencephaly (53%), unilateral overgrowth (40%), and autism spectrum disorder (27%) [5,6]. In relation to case 1 in this study, the symptoms of seizures, and intellectual delay were all present. The presence of hypopigmentation is related to pigmentary mosaicism, which describes an individual or tissue that contains two or more different cell lines typically derived from a single zygote and is the result of mitotic nondisjunction [7].

According to the study of the clinical spectrum of MTOR-related hypomelanosis of Ito with neurodevelopmental abnormalities by Carmignac et al., MTOR pathogenic variants were found in more than one-third (36.6%) of the patients. This MTOR, together with PI3K, forms a complex that responds to growth factors [8]. The presence of seizures in MTOR-related hypomelanosis of Ito suggests that hemimegalencephaly in an MTOR pathogenic variant might be more strongly associated with epilepsy than megalencephaly [5].

The presence of seizure and hypopigmented skin lesion in case 1 prompted consideration of hypomelanosis of Ito. A diagnostic evaluation is suggested by Ruiz-Maldonado et al. in which a diagnosis is made based on the presence of one major and one minor criterion or two minor criteria. The major criteria are as follows: (A) non-hereditary cutaneous hypopigmented linear streaks or patches involving more than two body segments appearing at birth or in the first months of life; (B) one or more neurological or musculoskeletal manifestations. The minor criteria consist of the following: (a) chromosomal anomalies; and (b) two or more congenital malformations, excluding malformations in the nervous and musculoskeletal systems [2,9].

In terms of the treatment of hypomelanosis of Ito, the symptoms related to the skin lesions usually do not require special treatment. The type of epilepsy should be established initially since they differ in drug tier. For the patient in case 1, a diagnosis of focal motor onset tonic-clonic seizures was established; hence, the patient was given 5 mg of oxcarbazepine at 300 mg/5 mL every 12 hours (22 mg/kg/day).

The patient in case 1 returned to the outpatient department for follow-up with their pediatrician. Following diagnosis, patients with this disease usually return to monitor their growth and development over time [10].

Linear nevus sebaceous syndrome

Case 2 presents an example of linear nevus sebaceous syndrome due to the presence of seizure, abnormal neurologic findings, and hyperpigmented skin lesions. Linear nevus sebaceous syndrome is a rare neurocutaneous syndrome that affects one in 1,000 live births [10] as a result of somatic mosaicism. This syndrome affects multiple organs, and patients with this syndrome present with a large facial nevus, which is usually located in the midline of the forehead and nose, neurodevelopmental abnormalities, and systemic defects [3].

Almost two-thirds of patients with linear nevus syndrome show neurologic symptoms [3], such as developmental delay, seizures, and hemiparesis. Cerebral and cranial anomalies, predominantly hemimegalencephaly and enlarged lateral ventricles, are reported in 72% of cases [3]. The incidence of epilepsy and intellectual disability is as high as 75% and 60%, respectively, in this syndrome [3]. In our case, the patient was noted to have seizures and developmental delays at seven months old. There were a variety of other cranial abnormalities, such as corpus callosum agenesis, agyria/microgyria, and pachygyria [11]. Pachygyria, hemimegalencephaly, and lateral ventricle enlargement were also present in the patient’s cranial MRI.

In terms of pathophysiology, linear nevus sebaceous syndrome is a result of a postzygotic RAS mosaicism mutation, such as in the HRAS (HRas proto-oncogene, GTPase), KRAS (KRAS proto-oncogene, GTPase), and NRAS (neuroblastoma RAS viral oncogene homolog) genes [12]. These RAS isoforms play a role in the regulation of cell survival, proliferation, and differentiation.

A study reported by Groesser et al. involving two linear nevus sebaceous syndrome patients and 63 patients with nevus sebaceous identified HRAS mutations in 95% of the cases and KRAS mutations in the remaining 5% of the cases [12]. A study by Chun Pan et al. reported consistent findings regarding postzygotic mutation through the identification of mutated KRAS genes in their index patient’s peripheral blood sample and non-lesional skin [13].

In terms of the central nervous system (CNS) abnormalities associated with this syndrome, unilateral hemimegalencephaly (HME) is the most common finding, as this is present in 50% of patients with linear nevus sebaceous syndrome; additionally, the HME is typically ipsilateral to the skin lesions [14]. However, the HME was contralateral in our case. Common MRI findings in HME are ventricular enlargement, increased signal intensity in the white matter of the affected hemisphere on T2, a loss of delineation between the white and gray matter, and agyria [9,10]. Additional work-up involving blood tests is typically unnecessary, with the exception of patients presenting with the above-mentioned symptoms [15].

Regarding the future prognosis, the risk of developing secondary carcinoma with linear nevus sebaceous syndrome is low, while the risk of developing secondary benign neoplasms is relatively high. In relation to the case presented in this work, the skin biopsy of the patient showed a non-malignant pathology.

Finally, the treatment of this syndrome is still symptom-based. In relation to the case, the patient’s seizures were medications resistant; hence, the patient was maintained on levetiracetam 100 mg/ml 2.6 ml twice per day (60 mg/kg/day), topiramate 43 mg/pptab once a day (OD) (5 mg/kg/day), and lacosamide 43 mg/pptab OD (5 mg/kg/day). The patient returned for follow-up appointments regularly on an outpatient basis, and seizure control was noted.

Comparison of hypomelanosis of Ito and linear nevus sebaceous syndrome

In this paper, both patients showed the presence of seizure, abnormal neurologic findings, cutaneous lesion, and developmental delay that prompted a diagnosis of neurocutaneous disease and underwent diagnostic workup. Both of their diagnostic workups showed hemimegalencephaly, voltage asymmetry, and basketweave orthokeratosis in skin biopsy. In magnified view, hypomelanosis of Ito skin biopsy showed decreased melanocytes and melanin at the basal layer while for the linear nevus sebaceous syndrome, it showed hypergranulosis with focal koilocytic change and hyperplasia with a flat base. In terms of pathophysiology, it has been shown that hypomelanosis of Ito and linear nevus sebaceous syndrome may be due to a mutated MTOR gene-producing signaling pathway, which plays a pivotal role in cell growth, protein synthesis, autophagy, and cystoskeletal dynamics [5] and the RAS isoform that is also responsible for controlling cell growth and survival, respectively [13].

## Conclusions

This paper aimed to discuss the similarities and differences between the two patients and the pathways that can be linked to the generation of the symptoms. The presence of early-onset seizures with no known history of fetal complications warrants meticulous physical examinations. The combination of a triad of intractable epilepsy, developmental delay, and cutaneous lesions should prompt consideration of a neuroectodermal disease. In this comparative study, these diseases may present similarly except for in terms of their pigmentation. Both diseases may involve pigmentary mosaicism, and the neurological findings could be due to the same pathway causing hemimegalencephaly and, subsequently, seizure and delay.

The diagnosis of these diseases is still clinical, and the treatment follows a symptomatic approach, especially for seizures. Moreover, the cutaneous lesions usually do not require special treatment except if the skin biopsy shows a high risk for the development of carcinoma.
